# Reading processes of public and private middle school and high school students

**DOI:** 10.1186/s41155-024-00296-0

**Published:** 2024-04-15

**Authors:** Adriana Marques de Oliveira, Jair Lício Ferreira Santos, Simone Aparecida Capellini

**Affiliations:** 1 Department of Speech and Hearing Sciences, Federal University of Santa Maria (Universidade Federal de Santa Maria – UFSM), Av. Roraima n° 1000 Cidade Universitária Bairro - Camobi, prédio 26 E, Santa Maria, Rio Grande do Sul 97105-900 Brazil; 2 Investigation Learning Disabilities Laboratory (LIDA), Department of Speech and Hearing, Sciences, São Paulo State University “Júlio de Mesquita, Filho” (UNESP), Av. Hygino Muzzi Filho, 737; Bairro: Mirante, São Paulo, Marilia CEP: 17.525-900 Brazil; 3 Department of Social Medicine, School of Medicine of Ribeirão Preto - University of São, Paulo (Universidade de São Paulo - USP), Av. dos Bandeirantes, 3900, Monte Alegre, São Paulo, Ribeirão Preto CEP 14040-900 Brazil; 4 Department of Speech and Hearing Sciences, São Paulo State University “Júlio de Mesquita, Filho” (UNESP), Marilia, São Paulo, Brazil

**Keywords:** Reading process, Evaluation, Adolescence, Elementary school, High school

## Abstract

**Introduction:**

Reading has been widely discussed, mainly due to the published results of international performance tests of schoolchildren. The gaps generated in literacy hinder the development of basic skills necessary for reading, which will have a negative impact on the teaching-learning process from elementary school to high school. This study aimed to compare the reading performance of the students in public and private schools through tests of the Brazilian reading processes-PROLEC-SE-R.

**Methods:**

Cross-sectional study. The Brazilian adaptation of the PROLEC-SE-R was administered to 436 students: 221 from the state school (G1 6th year, *n* = 30; G2 7th year, *n* = 33; G3 8th year, *n* = 35; G4 9th year, *n* = 31; G5 1st year, *n* = 32; G6 2nd year, *n* = 30; G7 3rd year, *n* = 30) e 215 private schools (G8 6th year, *n* = 31; G9 7th year, *n* = 31; G10 8th year, *n* = 30; G11 9th year, *n* = 31; G12 1st year, *n* = 30; G13 2nd year, *n* = 31; G14 3rd year, *n* = 31). Tools of descriptive and bivariate analysis were used.

**Results:**

Superior performance of the private school students on spelling tests helps their reading as evidenced by their scores for syntactic and semantic processes. When the knowledge of the use of the word in text, extraction of meaning and its understanding was needed, the difficulty of access to the mental lexicon of the studied population became evident.

**Conclusion:**

The PROLEC-SE-R, in addition to establishing the reading profile of elementary and high school students, shows that the gaps in teaching and learning, which exist between public and private education in the literacy period, accompany students throughout the basic education cycle. Knowing the reading profile and in which process there is a disruption is important so that the teaching of specific strategies can be promoted throughout the entire schooling process, especially in primary and secondary education.

## Introduction

Reading is widely recognized as the most crucial skill to acquire. It involves a complex process of extracting and constructing meaning from written language. Moreover, its impact on students’ lives extends to learning, cognitive development, academic achievements, success across various school subjects, and problem-solving abilities. Proficiency in reading comprehension forms the bedrock for success in all academic realms, from elementary through higher education (Barber et al., 2020; Danaei et al., 2020; Kao et al., 2016).

As per Larsen and Little (2023), a fundamental objective of school education is to equip students with reading skills that empower them to thrive in adulthood. Enhanced academic skills during childhood correlate with improved prospects in the job market (Hanushek et al., 2015; Parsons et al., 2011) and a higher socioeconomic status in adulthood (Ritchie & Bates, 2013). Reading skills are pivotal in the school curriculum, and competence in this area is a prerequisite for mastering the majority of subject matter throughout the compulsory school years and beyond. Students’ reading performance should advance with each passing year of education, influenced by both biological development and educational experience (Morrison et al., 2019).

Disruptions in the learning process can lead to academic and social setbacks in individuals’ lives. Consequently, it is of utmost importance for professionals working with learning and its challenges to comprehend the intricacies of reading and comprehension and intervene when any of these skills are compromised (Larsen & Little, 2023).

Reading is a highly complex skill that involves a variety of cognitive processes and forms of representation. Neuroimaging studies have identified an extensive network of crucial brain regions for proficient reading and the development of this skill. Effective reading is associated with several partially independent subskills. This includes decoding, where readers decipher unfamiliar words by relating orthographic representations to phonological and later semantic knowledge. It also encompasses sight word reading, where readers directly recognize familiar words through orthographic representations and semantic knowledge. Furthermore, comprehension is essential, as it involves connecting orthographic and phonological information to semantic knowledge, enabling the reader to understand a word or written text. Fluent reading also necessitates automatization in attention and perception (Arnell et al., 2009; Cross et al., 2023).

Given the importance of reading in the academic and social life of human beings, researchers from several countries have been concerned with the issues of reading problems and their understanding. This concern is evidenced in articles published by researchers from China, Spain and the United States, for example (Ahamed et al., 2016; Álvarez-Cañizo et al., 2020; Chung & Lam, 2020; Wexler et al., 2022).

The most recent results of the International Student Evaluation Program (PISA), coordinated in Brazil by the Anísio Teixeira National Institute of Educational Studies and Research (INEP) and conducted in 2018, were released in 2019 (Brazil, 2019; OECD, 2019). Overall, Brazil ranks 57th out of the three areas evaluated (reading, mathematics, and science). Specifically, in reading, the country is placed 58th. The percentage of students at level 2, considered by the OECD to be the minimum required for active citizenship, stands at 24.5%. In contrast, 50.1% fall below this level. This indicates that, for every 10 students, five fail to attain the necessary reading and comprehension skills. A total of 25.6% of students reach level 3 or above in reading, which demands critical thinking regarding text portions and the comparison of different authors’ viewpoints. Out of the 10,961 students evaluated, a mere 0.2% achieved the highest level of reading proficiency. According to PISA data, Brazil lags OECD countries in terms of schooling by approximately two and a half years (Brazil, 2019; OECD, 2019).

This could be reflected, as we can see in the study by Guarinello et al. (2023), which analyzed the perception of students from two higher education courses regarding their experiences and practices of reading and writing texts belonging to the discourse genre in the academic sphere. The authors identified that many students assume they have some difficulty in using the discursive genres used in the university, which may indicate gaps in the work with written language at the educational levels that preceded their entry into higher education.

In a study conducted by Oliveira and Capellini (2010), the reading processes of elementary school students in both public and private schools were assessed. The findings revealed that students attending private schools outperformed their public school counterparts in tests evaluating letter recognition, lexicon, syntax, and semantics. Of particular note was the remarkably low average score of public school students in phoneme knowledge, indicating a deficiency in teaching grapheme-phoneme relationships within public school classrooms.

The failure to prioritize phonemic awareness during the early stages of literacy instruction is a significant factor contributing to the literacy gap. These gaps in literacy underscore the deficiency in developing fundamental reading skills, such as building a robust mental lexicon and mastering the decoding process for word recognition (Álvarez-Cañizo et al., 2020; Cuetos, 2010; Oliveira, 2017). This deficiency has far-reaching consequences, negatively affecting the teaching and learning process from elementary to high school and even in higher education. The inability to recognize words, comprehend their relationships within sentences, and grasp the coherence between sentences can significantly hinder a profound understanding of the text (Álvarez-Cañizo et al., 2020; Cuetos, 2010; Guarinello et al., 2023; Silva & Pereira, 2019).

In a Brazilian study conducted in 2019 by Andrade, Celeste, and Alvez, researchers characterized reading fluency among students in Middle School. The results indicated that reading fluency and accuracy rates gradually increase as students’ progress through Middle School. According to the authors, measures of reading fluency reflect the students’ ability to comprehend the meaning of the text they read. However, the study did not identify significant differences when comparing performance between genders and among the surveyed schools.

Based on the above, the following question was raised: Is there a difference in the evaluation of reading processes between public and private elementary school students through tests of the Brazilian reading processes-PROLEC-SE-R?

The aim of this study was to compare the performance of students in elementary and secondary education in public and private schools through tests of the Brazilian reading processes-PROLEC-SE-R.

## Methods

### Design of the study

The cross-sectional approach was used to characterize and to compare the performance of students in elementary and secondary education in public and private schools through tests of the Brazilian reading processes-PROLEC-SE-R.

### Participants

A total of 436 students were randomly selected from the reference population, of which 221 (50.69%) were from public schools and 215 (49.31%) were from private schools. Of these, 263 were female (145 public education and 118 private education) and 173 were male (76 in public school and 97 in private school), subdivided into the following groups:

- 221 students from state public schools
  - G1: 30 from the 6th year of elementary school, mean age 11.2 years (SD: 0,48)
  - G2: 33 from the 7th year of elementary school, mean age 11.9 years (SD: 0,38)
  - G3: 35 from the 8th grade of elementary school, mean age 12.8 years (SD: 0,56)
  - G4: 31 from the 9th year of elementary school, mean age 13.9 years (SD: 0,59)
  - G5: 32 from the 1st year of high school, mean age 14.8 years (SD: 0,75)
  - G6: 30 from the 2nd year of high school, mean age 16.0 years (SD: 0,45)
  - G7: 30 from the 3rd year of high school, mean age 17.1 years (SD: 0,35)
- 215 private school students
  - G8: 31 from the 6th year of elementary school, mean age 11.2 years (SD: 0,40)
  - G9: 31 from the 7th year of elementary school, mean age 12.6 years (SD: 0,35)
  - G10: 30 from the 8th year of elementary school, mean age 12.9 years (SD: 0,64)
  - G11: 31 from the 9th year of elementary school, mean age 13.9 years (SD: 0,48)
  - G12: 30 from the 1st year of high school, mean age 15.1 years (SD: 0,44)
  - G13: 31 from the 2nd year of high school, mean age 16.2 years (SD: 0,54)
  - G14: 31 from the 3rd year of high school, mean age 17.2 years (SD: 0,56)

The selection criteria were as follows:

- Inclusion criteria were 1) parents or guardians signed an informed consent form; 2) they signed the Term of Assent; 3) children were regularly enrolled in elementary school cycle II or high school in the participating schools.
- Exclusion criteria were 1) students who refused to participate, although the parents or guardians signed the informed consent form; 2) students with an interdisciplinary diagnosis of learning disorder, dyslexia and attention deficit hyperactivity disorder; 4) learning difficulty; 5) alteration of language or speech; 6) impaired visual and auditory acuity; 7) diagnosis of genetic or neurological syndromes; 8) history of repetition; 9) intellectually compromised.

These criteria, except for the Informed Consent and Assent Form, were observed in the participants’ school records and/or reported by the teachers and school coordinators. All information related to learning complaints and diagnoses is included in the student’s record with reference to the ICD or DSM-V. Learning complaints reported by teachers, when not accompanied by documentation, were compared with school grades. The criterion adopted was to exclude students with an overall average below five.

Some students were excluded from the sample after data collection because language and speech impairments were detected during test administration. It is worth noting that all students who submitted the informed consent form and signed the assent form were assessed, despite the detected impairments, so that they would not feel excluded or exposed to their classmates. However, they do not constitute the sample of this study.

Data was collected in a central-western São Paulo city in 2016, with a population of approximately 233,639. The population had an average monthly salary of 2.5 minimum wages and a per capita income of 36,163.08 Brazilian reais (BRASIL, 2016a). During the study period, the city reported 42,696 enrolled students: 24,605 in Elementary School, 9976 in High School, 170 in Youth and Adult Education (EJA), 393 in Special Education, 9481 in Early Childhood Education (preschool and kindergarten), and 1672 (3.3%) in Vocational Education (Brasil, 2016a).

In the School Performance Evaluation (ANRESC) – PROVA BRASIL (Brazil Test), conducted by INEP (Brasil, 2016b), the municipality achieved an average score of 251.25 in Portuguese language (the national school average in Brazil was 251.53, and in the state of São Paulo, it was 257.37).

School 1 is a state-run public institution for Middle School education on a full-time basis, with 323 enrolled students in 2015. In addition to the regular Middle School curriculum, it provides study guidance, youth leadership, and values for civic life. The school carries out projects involving sports activities, reading encouragement, nature preservation, health awareness, and cultural initiatives. In the School Performance Evaluation - ANRESC - Prova Brasil of 2015, 97.85% of the students participated and achieved an average score of 278.96 in Portuguese Language and 285.39 in Mathematics. Regarding teacher training, the school obtained a rate of 61.10% (Brazil, 2016b).

School 2, a public institution, educates 845 students in Middle School, High School, and EJA. High School classes run in the morning, Middle School in the afternoon, and evenings cater to High School and EJA students. The school offers extracurricular activities like sports, reading, culture, and health programs. Collaborating with local universities, it engages in research, career guidance, and teacher training. Internships involve students in Psychology, Physical Education, History, Mathematics, and Sociology, benefiting both teachers and students. In 2015, 90.36% of 9th-grade students took part in the Prova Brasil, achieving averages of 258.64 in Portuguese and 265.34 in Mathematics. Teacher training rates were 51.40% for Middle School and 58.60% for High School (Brasil, 2016b; Brasil, 2016c). In the National High School Examination (ENEM), High School students achieved an average score of 481.00, with notable scores of 488.19 in Writing and 491.62 in Language, Codes, and Technologies (Brasil, 2016c).

School 3 is a state-run institution that offers Middle School and High School education. The morning period is dedicated to High School, while the afternoon is focused on Middle School. Currently, the school has a total of 703 enrolled students, with a maximum capacity of 40 students per classroom for Middle School and 45 students per classroom for High School. The school has partnerships with city universities for psychology internships and research. It offers extracurricular classes in crafts, chess, and music outside regular school hours. Regarding student performance, 91.51% of 9th-grade students took the Prova Brasil, with averages of 267.99 in Portuguese and 271.52 in Mathematics. The school’s teacher training is 46.90% for Middle School and 49.60% for High School (Brasil, 2016a; Brasil, 2016c). In the 2015 ENEM exam, 37 High School students (58.73%) achieved an overall score of 500.00, with averages of 501.79 in Languages, Codes, and Technologies and 540.00 in Writing (Brasil, 2016c).

School 4 is a private institution that covers Elementary and Middle School, High School, and Pre-Vestibular (college entrance exam preparation). Elementary School has classes in the morning and afternoon, while High School has classes in the morning and evening. With 1180 enrolled students, up to 45 students per classroom are allowed, except for the pre-vestibular and the 3rd year of High School. The school offers extracurricular activities, including ecological trips, socio-sports competitions, cultural events (dance, music, theater, and poetry), career fairs, interclass games, and visits to local colleges. In the ENEM (BRASIL, 2016c), the school achieved an average score of 564.00, with 604.79 in Writing and 553.89 in Languages, Codes, and Technologies. The reported teacher training indicator was 60.50.

### Instruments

The Brazilian Adaptation of the Reading Processes Assessment (PROLEC-SE-R) (Oliveira, 2017; Oliveira et al., 2020) aims to assess lexical, syntactic, and semantic reading processes. It consists of 13 tests, with the first six comprising the screening version, which can be administered collectively or individually. The materials included in the battery are: two test booklets; 1) the screening version of tests 1 to 6 (which the student has access to during the evaluation); and 2) tests 7 to 13 administered individually, along with the answer sheet. The tests are described as follows:

#### Lexical selection (LS)

Indicates the ability to recognize words without the need to access their meaning. It is a measure of accuracy and speed in word recognition. A list of 50 words, 25 real and 25 invented. The examinee should indicate which ones are real and which ones are invented.

#### Semantic categorization (CS)

Indicates the speed of access to the meaning of words. A list of 90 words, half of which represent animals and the other half do not. The examinee should indicate whether the word refers to an animal or not.

#### Grammatical structures I (GS II)

Indicates the ability to process syntactically complex sentences with different grammatical structures. There are 24 drawings accompanied by three sentences. The examinee should indicate which sentence accurately describes what is represented in the drawing.

#### Grammatical judgment (GJ)

Evaluates the ability to syntactically process sentences by detecting grammatically correct phrases within a two-minute period. There are 35 sentences, and the examinee should indicate which ones are grammatically correct and which ones are incorrect.

#### Expository comprehension (EC)

Indicates the ability to extract the message from expository text and integrate it into memory. Expository text with 10 questions, each having four answer options. No reference materials allowed, silent reading.

#### Narrative comprehension (CN)

Indicates the ability to extract the message and form a mental representation, in this case, of narrative texts. Narrative text with 10 questions, each having four answer options. Reference materials are allowed, silent reading.

#### Word Reading (WR)

Indicates the ability to retrieve word pronunciation based on written form. Reading aloud four word lists. Record the reading time, number of correct answers, and describe the errors made.

#### Pseudoword Reading (PR)

Indicates the use of the phonological route, i.e., the ability to use grapheme-to-phoneme conversion rules. Reading aloud two lists of pseudowords. Record the reading time, number of correct answers, and describe the errors made.

#### Grammatical structures II (GSII)

Indicates the ability to syntactically process grammatically complex sentences with different grammatical structures, each with only one sentence to analyze. There are 24 items, each with four drawings and one sentence. Identify which of the four drawings corresponds to the sentence. Record the response and the number of correct answers.

#### Punctuation Marks (SP)

Indicates the ability to respect punctuation marks. Narrative text. Reading aloud. Record the correctly read punctuation marks and note the errors.

#### Pure Reading comprehension (PCR)

Indicates the ability to understand expository texts without the influence of memory. Expository text. Reading aloud with 10 questions. Record reading time and answers to the questions. Reference to the text is allowed. Note correct and incorrect answers.

#### Mnemonic Reading comprehension (MRC)

Indicates the ability to comprehend expository texts with the influence of memory. Expository text. Silent reading with 10 questions without consulting the text. Note correct and incorrect answers.

#### Listening comprehension (LC)

Indicates the ability to comprehend a text without the influence and intervention of reading. Expository text. The evaluator reads aloud twice and asks 10 questions to the student. Record correct and incorrect answers.

### Procedures

The tests were individually administered by the researcher either in a classroom provided by the school or in the reading room during the student’s regular class period. To remove students from their class, prior permission was requested from the teacher. The students’ departure was contingent upon the authorization of the responsible teacher and the content being taught at the time.

The answer sheet remained in the possession of the evaluator, along with a stopwatch and pencils for note-taking, with an average duration of 40 minutes for its completion. The following procedures were adopted:

- Signing of the Free and Informed Consent Form by the guardians
- Signature of the Term of Assent by the students
- Survey of Portuguese and all subjects, except physical education
- Application of the Reading Processes Assessment Tests-PROLEC-SE-R, individual version (Oliveira, 2017; Oliveira et al., 2020).

For this study, tests 7 to 13 from the individual version were used, in the following order:

- Word Reading: The purpose of this test is to understand the functioning of lexical and phonological word recognition routes. This task consists of reading four lists of words aloud (WR1 to WR4). Each list contains 24 words, distributed as follows: WR1 short- and high-frequency words, WR2 long- and high-frequency words, WR3 short- and low-frequency words and WR4 long- and low-frequency words. The time spent reading should be noted.
- Pseudoword reading: The purpose of this test is to understand the functioning of lexical and phonological word recognition routes. This task consists of reading the two lists (PR1 and PR2) aloud. The pseudowords were divided into short (disyllabic pseudowords - PR1) and long (trisyllabic and polysyllabic pseudowords - PR2). The time spent reading should be noted.
- Grammatical Structures II (GSII): The purpose of this test is to assess the ability to process sentences with different types of grammatical structures. In this test, the task is to identify the drawing that corresponds to that indicated by the sentence. It contains 24 stimuli and an example.
- Punctuation Marks (PM): The purpose of this test is to assess the correct intonation of the marked punctuation marks. In this test, the text *“Maldito apêndice”* (Cursed Appendix) is presented for reading aloud. The examiner should pay attention to the correct intonation of the marked punctuation marks, noting an ‘x’ in the spaces provided next to each mark only in case of intonation errors. There are a total of 31 punctuation marks.
- Pure Reading Comprehension (PRC): The objective is to assess the student’s ability to comprehend expository text without the interference of memory. The reading will be conducted aloud, and the time should be recorded. The text is expository with 10 literal and inferential questions. The student can consult the text to answer the questions.
- Mnemonic reading comprehension (MRC): This test evaluates the student’s ability to understand expository texts with memory interference, with open questions. The task consists of reading the text in silence. The text is expository, and the 10 questions are literal. The student cannot consult the text to answer the questions.
- Listening Comprehension (LC): In this test, the examiner read a text to the student twice, aloud. Then, one by one, the 10 questions are asked.

## Data analysis

A database was created in a Microsoft Excel spreadsheet and then transferred to STATA/SE (version 13.1) for statistical analysis. Descriptive statistical tools were used to characterize the sample. To assess whether one average was higher than the other concerning variables such as time and grades, stratified by school year and type of education, was conducted *Student’s T-Test*. Furthermore, were calculated 95% confidence intervals for mean estimates using Student’s t-distributions. To compare students’ performance in the PROLEC-SE-R tests based on the type of education (public and private), was applied the Wilcoxon test (Mann–Whitney test).

Except for Table 1 – which refers to socio-demographic and educational variables (such as age and grade) – all the others underwent multiple comparisons, in addition to the usual 2 × 2 tests. Multiple comparisons were applied in each of the sections of the tables with 7 comparisons of the variables. The Holm (1979) was used. In this application, recorded values such as < 0.001 or 0.000 were considered as 0.0005.

**Table 1:** Distribution of the average age by school grade, for each type of education, and description of the execution time in minutes of the individual version tests for middle school and high school students

	Group	Mean (time)	SD	95%CI		*p*-value
**AGE**	**Elementary school II**					
	G1	11.20	0.48	11.01	11.38	0.522
	G8	11.19	0.40	11.04	11.34	
	Total	11.19	0.44	11.08	11.30	
	G2	11.90	0.38	11.77	12.04	0.050
	G9	12.06	0.35	11.93	12.19	
	Total	11.98	0.37	11.89	12.07	
	G3	12.82	0.56	12.63	13.02	0.487
	G10	12.83	0.64	12.59	13.07	
	Total	12.83	0.60	12.68	12.97	
	G4	13.90	0.59	13.68	14.12	0.321
	G11	13.96	0.48	13.79	14.14	
	Total	13.93	0.53	13.79	14.07	
	**High school**					
	G5	14.78	0.75	14.51	15.05	0.038*
	G12	15.06	0.44	14.89	15.23	
	Total	14.91	0.63	14.75	15.08	
	G6	16.00	0.45	15.83	16.16	0.069
	G13	16.19	0.54	15.99	16.39	
	Total	16.09	0.50	15.96	16.22	
	G7	17.13	0.35	17.00	17.26	0.221
	G14	17.22	0.56	17.02	17.43	
	Total	17.18	0.46	17.06	17.29	
**INDIVIDUAL VERSION – EXECUTION TIME**	**Elementary school II**					
	G1	36.46	5.30	34.48	38.44	0.495
	G8	36.48	5.37	34.51	38.45	
	Total	36.47	5.29	35.11	37.83	
	G2	35.15	6.84	32.72	37.57	0.544
	G9	34.96	6.27	32.66	37.26	
	Total	35.06	6.52	33.43	36.69	
	G3	33.37	5.96	31.32	35.41	0.238
	G10	34.56	7.47	31.77	37.35	
	Total	33.92	6.67	32.26	35.57	
	G4	35.38	6.14	33.13	37.63	0.999
	G11	30.61	4.02	29.13	32.08	
	Total	33.00	5.68	31.55	34.44	
	**High school**					
	G5	34.31	5.66	32.27	36.35	0.739
	G12	33.50	4.06	31.98	35.01	
	Total	33.91	4.93	32.66	35.17	
	G6	31.03	5.99	28.79	33.27	0.025*
	G13	33.70	4.31	32.12	35.29	
	Total	32.39	5.33	31.02	33.76	
	G7	31.50	4.79	29.71	33.28	0.360
	G14	31.90	3.91	30.46	33.34	
	Total	31.70	4.33	30.59	32.81	

Student’s t test. *Evidence of statistical association (*p* < 0.05)

## Results

To compare the average ages by school grade, for public and private schools, the *Student’s T-Test* and the 95% confidence interval (CI95%) were employed. The *Student’s T-Test* assessed whether one mean exceeded the other, while the CI 95% indicated the concentration of variability around the estimated value. The *Student’s T-Test* detected evidence that, for the 1st grade of high school, one mean surpassed the other. Upon analyzing the mean values obtained, it was observed that private school students had a higher average age than public school students. Despite this indication, when analyzing the CI 95%, it was found that the confidence intervals overlapped, suggesting equality between the means if the test were two-tailed.

To describe and compare the means of the time variables in the individual versions of PROLEC-SE-R, were conducted a Student’s T-Test. Were compared students by school year for each type of education. Evidence was found that the mean time in minutes is higher for private school students in the 2nd grade (public: ($\overline{x}$ = 31.03, *SD* = 5.99; private: $\overline{x}$ = 33.70, *SD* = 4.31, *p* = 0.025). The significance level adopted was *p* < 0.05. The value *p* = 0.025 represents the probability of obtaining the observed results if the null hypothesis were true, indicating a statistically significant difference between the mean time of public and private school students in the 2nd grade of high school.

Regarding the Portuguese grades (Table 2) and the average grades across all school subjects, except physical education, there is evidence that the average for the 6th year in private education is higher than in public education (*p* = 0.009*, public: $\overline{x}$ = 6.70, *SD* = 1.45, 95% *CI*: 6.15; 7.24; private: $\overline{x}$ = 7.57, *SD* = 1.32, 95% *CI*: 7.03; 8.05). After applying the Bonferroni multiple comparison method to analyze the mean scores, no statistical evidence of difference between them was found. This suggests that, while statistically significant differences were initially detected, they did not remain significant after adjusting for multiple comparisons. This analysis influences the interpretation of the data by suggesting that the observed differences in mean scores may have arisen by chance, due to random variation, and may not necessarily represent genuine differences between the compared groups.

**Table 2:** Distribution of Portuguese grades and overall average grades from all 1st semester courses

	Group	Mean (grade)	SD	95% CI		*p*-value	Multiple comparisons	
							*p-*value	significance
**Portuguese score**	**Elementary school II**							
	G 1	6.70	1.45	6.15	7.24	0.009*	0.007	no
	G 8	7.57	1.32	7.03	8.05			
	Total	7.14	1.45	6.77	7.51			
	G 2	6.83	1.72	6.22	7.44	0.114	0.010	no
	G 9	7.29	1.27	6.82	7.76			
	Total	7.05	1.53	6.67	7.44			
	G 3	7.30	1.39	6.81	7.78	0.112	0.008	no
	G 10	7.66	0.91	7.32	8.00			
	Total	7.46	1.20	7.17	7.76			
	G 4	6.50	1.93	5.79	7.21	0.509	0.013	no
	G 11	6.49	1.27	6.02	6.96			
	Total	6.49	1.62	6.08	6.91			
	**High school**							
	G 5	6.70	1.83	6.03	7.36	0.947	0.017	no
	G 12	6.11	0.69	5.85	6.37			
	Total	6.41	1.42	6.05	6.78			
	G 6	7.39	1.32	6.90	7.89	1.000	0.025	no
	G 13	5.80	1.12	5.39	6.21			
	Total	6.58	1.45	6.21	6.96			
	G 7	8.21	1.20	7.76	8.66	1.000	0.050	no
	G 14	5.82	1.23	5.37	6.27			
	Total	7.00	1.70	6.56	7.43			
**Overall average score**	**Elementary school II**							
	G 1	7.18	1.36	6.67	7.69	0.132	0.007	no
	G 8	7.56	1.30	7.08	8.04			
	Total	7.37	1.33	7.03	7.72			
	G 2	7.74	1.21	7.31	8.17	0.952	0.013	no
	G 9	7.19	1.38	6.68	7.69			
	Total	7.47	1.31	7.14	7.80			
	G 3	7.24	1.45	6.74	7.74	0.193	0.008	no
	G 10	7.51	0.96	7.15	7.87			
	Total	7.37	1.25	7.05	7.68			
	G 4	6.85	1.59	6.27	7.44	0.723	0.010	no
	G 11	6.63	1.37	6.12	7.13			
	Total	6.74	1.47	6.36	7.11			
	**High school**							
	G 5	7.01	1.16	6.60	7.43	0.999	0.017	no
	G 12	5.93	0.90	5.59	6.27			
	Total	6.49	1.17	6.19	6.79			
	G 6	7.48	1.19	7.04	7.93	1.000	0.025	no
	G 13	6.18	1.18	5.74	6.61			
	Total	6.82	1.34	6.47	7.17			
	G 7	7.06	1.02	6.68	7.44	1.000	0.050	no
	G 14	5.52	1.21	5.07	5.96			
	Total	6.28	1.36	5.93	6.63			

*Student’s t test*

*Bonferroni multiple comparison*

*Evidence of statistical association (*p* < 0.05)

In word reading list 1 (Table 3), evidence of a difference in the mean of correct answers was observed for 7th graders (*p =* 0.025); however, no evidence of a difference in time in seconds was found for this list.

**Table 3:** Description and comparison of correct answers in Word Reading tests 1, 2, 3 and 4

Group					Multiple Comparisons	
	Mean	SD	Median	*p-*value	*p-*value	significance
PL 1						
G 1	23.63	0.61	24.00	0.487	0.050	no
G 8	23.74	0.51	24.00			
Total	23.68	0.56	24.00			
G 2	23.54	0.51	24.00	0.025*	0,007	no
G 9	23.87	0.34	24.00			
Total	23.70	0.55	24.00			
G 3	23.80	0.40	24.00	0.124	0.008	no
G 10	23.93	0.25	24.00			
Total	23.86	0.34	24.00			
G 4	23.64	0.66	24.00	0.439	0.025	no
G 11	23.80	0.40	24.00			
Total	23.72	0.54	24.00			
G 5	23.81	0.39	24.00	0.332	0.017	no
G 12	23.90	0.43	24.00			
Total	23.85	0.35	24.00			
G 6	23.86	0.43	24.00	0.282	0.013	no
G 13	23.96	0.17	24.00			
Total	23.91	0.33	24.00			
G 7	23.93	0.25	24.00	0.145	0.010	no
G 14	23.80	0.40	24.00			
Total	23.86	0.34	24.00			
PL 2						
G 1	23.56	0.85	24.00	0.608	0.017	no
G 8	23.54	0.76	24.00			
Total	23.55	0.80	24.00			
G 2	23.45	1.03	24.00	0.045*	0.008	no
G 9	23.77	0.92	24.00			
Total	23.60	0.98	24.00			
G 3	23.77	0.49	24.00	0.256	0.010	no
G 10	23.90	0.30	24.00			
Total	23.83	0.41	24.00			
G 4	23.90	0.39	24.00	0.974	0.050	no
G 11	23.95	0.24	24.00			
Total	23.91	0.32	24.00			
G 5	23.71	0.58	24.00	0.029*	0.007	no
G 12	23.96	0.18	24.00			
Total	23.83	0.45	24.00			
G 6	23.90	0.18	24.00	0.966	0.025	no
G 13	23.90	0.30	24.00			
Total	23.90	0.30	24.00			
G 7	23.96	0.18	24.00	0.309	0.013	no
G 14	24.00	0.00	24.00			
Total	23.98	0.12	24.00			
PL 3						
G 1	22.43	2.32	23.00	0.820	0.050	no
G 8	22.77	1.54	23.00			
Total	22.60	1.96	23.00			
G 2	22.78	2.21	23.00	0.455	0.013	no
G 9	23.25	1.26	24.00			
Total	23.01	1.82	24.00			
G 3	23.57	0.65	24.00	0.154	0.007	no
G 10	23.30	0.87	23.00			
Total	23.44	0.77	24.00			
G 4	23.41	0.92	24.00	0.586	0.017	no
G 11	23.51	0.92	24.00			
Total	23.46	0.91	24.00			
G 5	23.21	1.66	24.00	0.615	0.025	no
G 12	23.60	0.77	24.00			
Total	23.40	1.31	24.00			
G 6	23.56	0.77	24.00	0.261	0.008	no
G 13	23.80	0.40	24.00			
Total	23.68	0.62	24.00			
G 7	23.76	0.50	24.00	0.398	0.010	no
G 14	23.58	0.84	24.00			
Total	23.67	0.70	24.00			
PL 4						
G 1	21.56	2.71	22.50	0.112	0.010	no
G 8	22.29	2.45	23.00			
Total	21.93	2.58	23.00			
G 2	22.06	1.91	22.00	0.037*	0.008	no
G 9	22.96	1.37	23.00			
Total	22.50	1.72	23.00			
G 3	22.94	1.32	23.00	0.137	0.013	no
G 10	23.43	0.93	24.00			
Total	23.16	1.18	24.00			
G 4	22.96	0.91	23.00	0.007*	0.007	no
G 11	23.48	1.02	24.00			
Total	23.22	0.99	24.00			
G 5	22.84	1.95	24.00	0.154	0.017	no
G 12	23.56	0.72	24.00			
Total	23.19	1.52	24.00			
G 6	23.53	0.86	24.00	0.436	0.050	no
G 13	23.74	0.51	24.00			
Total	23.63	0.70	24.00			
G 7	23.70	0.70	24.00	0.232	0.025	no
G 14	23.90	0.30	24.00			
Total	23.80	0.54	24.00			

Wilcoxon test

*Bonferroni multiple comparison*

*Evidence of statistical association (*p* < 0.05)

For word list 2 (Table 3), the *Wilcoxon Test* indicated evidence of a difference in correct answers for 7th graders (*p =* 0.045) and 1*st* graders (*p =* 0.029), while also showing a difference in mean time in seconds for 2*nd* graders (public: *x̅* = 19.43, *SD* = 5.27; private: *x̅* = 16.83, *SD* = 3.53, *p =* 0.024).

For word reading 3 (Table 3), no evidence of a difference in the average score of correct answers was found. However, there is evidence of a difference in mean time in seconds for the 6th year (public: $\overline{\ x}$ = 25.76, *SD* = 7.72; private: $\overline{x}$ = 22.70, *SD* = 9.67, *p* = 0.027), 9th year (public: $\overline{x}$ = 20.96, *SD* = 4.87; private: $\overline{x}$ = 17.32, *SD* = 3.59, *p* = 0.004), 1st grade (public: $\overline{x}$ = 21.62, *SD* = 5.10; private: $\overline{x}$ = 18.06, *SD* = 4.03, *p* = 0.005), and 2nd grade (public: $\overline{x}$ = 18.50, *SD* = 4.03; private: $\overline{\ x}$ = 16.32, *SD* = 3.54, *p* = 0.017).

In word list 4 (Table 3), differences were found for the 7th year (*p* = 0.037) and 9th year (*p* = 0.007) regarding the mean number of correct answers. Additionally, differences were observed in mean time in seconds for the 6th year (public: $\overline{\ x}$ = 41.16, *SD* = 14.11; private: $\overline{x}$ = 35.61, *SD* = 15.65, *p* = 0.028), 9th year (public: $\overline{x}$ = 31.12, *SD* = 6.61; private: $\overline{x}$ = 24.41, *SD* = 5.35, *p* < 0.001), 1st grade (public: $\overline{x}$ = 30.46, *SD* = 8.28; private: $\overline{x}$ = 24.10, *SD* = 4.12, *p* = 0.001), and 2nd grade (public: $\overline{x}$ = 27.00, *SD* = 5.30; private: $\overline{\ x}$ = 22.22, *SD* = 4.80, *p* < 0.001).

Upon applying the Bonferroni multiple comparison method to analyze the data, the previously observed differences were no longer evident. This finding suggests that while initial analyses indicated significant discrepancies between groups, these disparities did not withstand adjustment for multiple comparisons.

In relation to the reading of pseudowords (Table 4), no significant differences were observed across school years or between types of education regarding the mean number of correct answers. These findings suggest that the average performance of students in reading both short and long pseudowords remains consistent regardless of school year or educational setting.

**Table 4:** Description and comparison of correct answers in the reading test of Pseudowords 1 and 2 and of the syntactic process tests grammatical structures and punctuation marks

Group					Multiple comparisons	
	Mean	SD	Median	*p -*value	*p-*value	significance
LPS 1 - hits						
G 1	21.90	2.42	22.50	0.639	0.025	no
G 8	22.22	2.02	23.00			
Total	22.06	2.22	23.00			
G 2	22.27	2.51	23.00	0.780	0,050	no
G 9	22.41	1.94	23.00			
Total	22.34	2.24	23.00			
G 3	22.40	1.81	23.00	0.050	0.008	no
G 10	23.10	1.49	24.00			
Total	22.72	1.70	23.00			
G 4	22.93	1.54	24.00	0.430	0.013	no
G 11	22.64	1.74	23.00			
Total	22.79	1.64	23.00			
G 5	22.34	2.47	23.00	0.167	0.010	no
G 12	23.03	1.49	24.00			
Total	22.67	2.07	23.00			
G 6	22.40	1.97	23.00	0.038	0.070	no
G 13	23.25	1.06	23.00			
Total	22.83	1.62	23.00			
G 7	23.13	0.93	23.00	0.512	0.017	no
G 14	23.12	1.43	24.00			
Total	23.13	1.20	23.00			
LPS 2 - hits						
G 1	19.20	4.45	21.00	0.499	0.008	no
G 8	20.35	3.03	21.00			
Total	19.78	3.81	21.00			
G 2	20.60	3.72	22.00	0.956	0.050	no
G 9	20.93	3.16	22.00			
Total	20.76	3.43	22.00			
G 3	21.31	2.43	22.00	0.883	0.025	no
G 10	21.43	2.41	22.00			
Total	21.36	2.40	22.00			
G 4	21.19	2.74	22.00	0.869	0.017	no
G 11	21.45	2.24	22.00			
Total	21.32	2.49	22.00			
G 5	21.09	3.56	23.00	0.333	0.007	no
G 12	22.30	1.85	23.00			
Total	21.67	2.91	23.00			
G 6	22.13	2.25	22.50	0.625	0.013	no
G 13	22.06	1.84	23.00			
Total	22.09	2.03	23.00			
G 7	21.90	2.52	22.50	0.546	0.010	no
G 14	21.61	2.40	22.00			
Total	21.75	2.44	22.00			
Grammatical structures						
G 1	16.00	3.68	17.00	0.782	0.050	no
G 8	16.25	2.73	16.00			
Total	16.13	3.21	16.00			
G 2	16.87	2.38	17.00	0.467	0.017	no
G 9	17.58	2.59	17.00			
Total	17.21	2.49	17.00			
G 3	17.42	2.35	18.00	0.005*	0.007	yes
G 10	19.30	2.38	19.00			
Total	18.29	2.52	19.00			
G 4	17.38	2.87	17.00	0.013*	0.008	no
G 11	19.06	2.12	18.00			
Total	18.22	2.64	19.00			
G 5	17.15	2.51	17.00	0.032*	0.010	no
G 12	18.66	2.82	19.00			
Total	17.88	2.75	17.50			
G 6	17.96	2.05	18.00	0.057	0.013	no
G 13	19.19	2.61	19.00			
Total	18.59	2.41	19.00			
G 7	18.46	2.64	18.00	0.722	0.025	no
G 14	18.61	3.15	19.00			
Total	18.54	2.89	18.00			
Punctuation marks						
G 1	29.38	2.13	30.00	0.128	0.013	no
G 8	28.18	3.20	29.00			
Total	28.85	3.04	30.00			
G 2	28.18	3.20	29.00	< 0.001*	0.007	yes
G 9	29.93	2.64	31.00			
Total	29.03	3.05	30.00			
G 3	27.88	3.44	29.00	0.000*	0.008	yes
G 10	30.46	1.16	31.00			
Total	29.07	2.93	30.00			
G 4	28.25	3.28	30.00	< 0.001*	0.010	yes
G 11	30.67	0.54	31.00			
Total	29.46	2.63	30.50			
G 5	29.46	1.77	30.00	0.686	0.050	no
G 12	29.63	1.62	30.00			
Total	29.54	1.69	30.00			
G 6	29.73	2.01	30.50	0.165	0.017	no
G 13	30.03	2.02	31.00			
Total	29.88	2.00	31.00			
G 7	29.76	1.75	30.00	0.635	0.025	no
G 14	29.77	1.70	31.00			
Total	29.77	1.71	30.00			

Wilcoxon test

*Bonferroni multiple comparison*

*Evidence of statistical association (*p* < 0.05)

Evidence of differences in time in seconds was suggested for the pseudoword list 1 across the school years: 6th year (public: $\overline{\ x}$ = 32.10, *SD* = 8.27; private: $\overline{x}$ = 25.45, *SD* = 6.19, *p* = 0.002), 1st grade (public: $\overline{x}$ = 28.34, *SD* = 9.01; private: $\overline{x}$ = 21.43, *SD* = 4.06, *p* < 0.001), 2nd grade (public: $\overline{x}$ = 23.73, *SD* = 5.10; private: $\overline{x}$ = 20.29, *SD* = 5.23, *p* = 0.010), as well as for the pseudoword list 2: 6th year (public: $\overline{x}$ = 52.00, *SD* = 14.03; private: $\overline{\ x}$ = 43.45, *SD* = 12.32, *p* = 0.014), 7th year (public: $\overline{x}$ = 46.63, *SD* = 13.70; private: $\overline{x}$ = 39.45, *SD* = 5.91, *p* = 0.031), 9th year (public: $\overline{\ x}$ = 43.77, *SD* = 10.50; private: $\overline{\ x}$ = 35.09, *SD* = 7.44, *p* < 0.001), 1st grade (public: $\overline{x}$ = 42.71, *SD* = 11.42; private: $\overline{\ x}$ = 31.90, *SD* = 7.20, *p* < 0.001) and 2nd grade (public: $\overline{x}$ = 38.83, *SD* = 8.04; private: $\overline{x}$ = 32.83, *SD* = 5.75, *p* = 0.002). These findings indicate differences in reading speed between public and private school students across different grade levels and pseudoword lists.

By the average score, both for word reading and pseudoword reading, in the lists where evidence of a difference was found, the average for private school students was higher than that of public school students. However, regarding the average time in seconds, it is observed that public school students have a longer average time for reading when compared to private school students.

Regarding the assessment tests of the syntactic process (shown in Table 4), evidence of difference was found in the GS II test for the 8th grade (*p* = 0.005), 9th grade (*p* = 0.013) and 1st grade (*p* = 0.032). In the test, PM, mean of correct answers, for the 7th year (*p* < 0.001), 8th year (*p* = 0.000), and 9th year (*p* < 0.001).

Upon utilizing the Bonferroni multiple comparison method, no significant difference was found in the assessment of grammatical structure between the 9th grade of Elementary School II and the 1st grade of High School.

Regarding the evaluation of the semantic process (Table 5), in the PRC test, there was evidence of a difference regarding the number of correct answers for the 6th year (*p* = 0.045), 7th year (*p* = 0.003), 8th year (*p* = < 0.001) and 1st grade (*p* = 0.013). According to the average score, in the years in which there was evidence of difference, there was a superior performance of private school students compared to those in public school.

**Table 5:** Description and comparison of correct answers in the semantic process tests: Pure Reading Comprehension, Mnemonic reading comprehension and Listening Comprehension

Group	Mean	SD	Median	*p*-value	Multiple Comparisons	
					*p*-value	Significance
**PRC**						
G 1	3.00	1.92	3.00	0.045*	0.013	no
G 8	4.19	2.15	4.00			
Total	3.60	2.11	4.00			
G 2	3.57	1.85	3.00	0.003*	0.008	yes
G 9	5.06	1.91	5.00			
Total	4.29	2.01	4.00			
G 3	3.60	1.78	4.00	< 0.001*	0.007	yes
G 10	5.36	1.71	6.00			
Total	4.41	1.95	5.00			
G 4	4.00	2.08	4.00	0.257	0.025	no
G 11	4.64	2.00	4.00			
Total	4.32	2.05	4.00			
G 5	4.03	2.34	4.00	0.013*	0.010	no
G 12	5.33	1.86	5.50			
Total	4.66	2.20	4.00			
G 6	4.66	1.88	5.00	0.095	0.017	no
G 13	5.41	1.82	6.00			
Total	5.04	1.87	5.00			
G 7	4.63	2.00	5.00	0.468	0.050	no
G 14	5.19	1.72	5.00			
Total	4.91	1.87	5.00			
**MRC**						
G 1	2.66	2.30	2.00	0.002*	0.017	yes
G 8	4.61	2.33	5.00			
Total	3.65	2.50	3.00			
G 2	3.57	2.43	3.00	< 0.001*	0.007	yes
G 9	5.77	1.82	6.00			
Total	4.64	2.41	4.00			
G 3	4.25	2.21	4.00	< 0.001*	0.008	yes
G 10	6.33	1.86	6.00			
Total	5.21	2.29	5.00			
G 4	3.74	2.60	3.00	0.000*	0.010	yes
G 11	6.93	2.42	7.00			
Total	5.33	2.96	5.50			
G 5	4.46	3.02	4.50	0.015*	0.025	yes
G 12	6.43	1.95	6.50			
Total	5.41	2.73	6.00			
G 6	5.16	2.73	5.50	< 0.001*	0.013	yes
G 13	7.41	1.82	8.00			
Total	6.31	2.55	7.00			
G 7	6.33	2.00	6.00	0.162	0.050	no
G 14	6.74	3.06	8.00			
Total	6.54	2.58	7.00			
**LC**						
G 1	2.66	2.32	2.50	0.004*	0.007	yes
G 8	4.41	2.23	4.00			
Total	3.55	2.42	3.00			
G 2	4.09	2.68	4.00	0.180	0.025	no
G 9	4.96	2.45	5.00			
Total	4.51	2.59	4.00			
G 3	3.88	2.63	3.00	0.000*	0.008	yes
G 10	6.80	1.84	7.00			
Total	5.23	2.71	6.00			
G 4	3.70	2.11	3.00	0.002*	0.010	yes
G 11	5.83	2.70	6.00			
Total	4.77	2.63	5.00			
G 5	4.06	2.58	4.00	0.004*	0.013	yes
G 12	6.03	2.05	6.00			
Total	5.01	2.53	5.00			
G 6	5.13	2.99	5.50	0.127	0.017	no
G 13	6.32	2.28	7.00			
Total	5.73	2.70	6.00			
G 7	5.73	2.25	6.00	0.286	0.050	no
G 14	6.29	2.01	7.00			
Total	6.01	2.14	6.00			

Wilcoxon test

*Bonferroni multiple comparison*

*Evidence of statistical association (*p* < 0.05)

After applying the Bonferroni multiple comparison method to analyze the mean scores, no statistical evidence of difference between the 6th year and 1st grade was found. This suggests that, while statistically significant differences were initially detected, they did not remain significant after adjusting for multiple comparisons.

In MRC, there was evidence of a difference for all school years; this indicated a higher performance among private school students, except for 3rd grade (*p* = 0.162). Conversely, in the LC test, evidence of a difference was found only among the 6th graders (*p* = 0.004), 8th graders (*p* = 0.000), 9th graders (*p* = 0.002) and 1st graders (*p* = 0.004). Based on the average score, the performance of private school students is higher than that of public school students.

## Discussion

When analyzing the average age of the students, no difference was identified between the groups, except for the 1st year of high school. This can be explained by the variables such as school failure, late enrollment, diagnoses of learning disorders, and/or other comorbidities being controlled, which makes it difficult to differentiate ages and grades. According to INEP (Brazil, 2019), the age-grade gap represents a significant issue as many students are not in the appropriate school grade for their age, a finding supported by other national studies (Fritsch et al., 2014; Sampaio & Guimarães, 2009).

Regarding the average grades of Brazilian students in the Portuguese subject in the 1st semester and the overall average of all subjects, differences between types of education were observed only for the 6th grade. This discrepancy was also not found in the study conducted by Oliveira (2017), which compared the grades of elementary school students in Portuguese with those in public and private high schools. This consistency in results likely resulted from controlling the variables described in the inclusion and exclusion criteria.

However, according to the study conducted by Sampaio and Guimarães (2009), which compared the academic performance of students in public and private secondary schools, it was revealed that public school students attained lower grades compared to their private school counterparts. Additionally, private school students demonstrated the highest level of academic efficiency, followed by federal public and state public school students.

Based on the study’s findings, there is no statistical evidence of differences in the accuracy of reading between high-frequency and low-frequency words, as well as pseudowords. However, there is evidence indicating that the time taken to identify words is longer for students in public schools. This finding aligns with existing literature, which suggests that public school students encounter challenges in decoding words relying on phonological processing and applying orthographic rules (Oliveira, 2017; Oliveira & Capellini, 2010; Oliveira & Capellini, 2013; Psyridou et al., 2018; Silva & Pereira, 2019). Conversely, private school students may exhibit a more effective understanding of the grapheme-phoneme conversion mechanism. Additionally, research suggests that starting from the 4th grade, mental word representation contributes to precise decoding. Nevertheless, there is a lack of studies examining the performance of elementary and high school students in these areas comprehensively. Discrepancies between public and private school students are particularly observed at the word level, especially in dealing with long, infrequent words, whereas spelling aids reading comprehension. Longer pseudowords also present greater reading challenges, especially through the phonological route (Gonçalves et al., 2013; Oliveira & Capellini, 2013; Oliveira et al., 2016; Perfetti & Hart, 2002; Pontes et al., 2013; Sha & Woore, 2021).

Proficiency in phonological decoding, a crucial aspect of the phonological route, plays a pivotal role in vocabulary learning and expansion. It strengthens the connections between spoken and written word forms, thereby facilitating the development of robust lexical representations (Perfetti & Hart, 2002; Sha & Woore, 2021). National studies involving elementary school students consistently indicate that students attending private schools outperform their counterparts in public schools in assessments of reading and writing skills (Gonçalves et al., 2013; Oliveira & Capellini, 2013; Oliveira et al., 2016; Pontes et al., 2013).

In tasks involving syntactic and semantic processes, automating basic reading processes becomes essential to allocate cognitive resources towards comprehension and extracting meaning. However, while foundational, these processes alone are insufficient. The systematic teaching of steps such as understanding the relationships between words, sentence structure, coherence between sentences, lexical richness, prior knowledge, familiarity with the subject matter, and the macro and microstructure of the text, including critical-reflexive reflection on written material, is crucial. With experience, these skills develop and improve over time (Álvarez-Cañizo et al., 2020; Capellini et al., 2014; Cuetos, 2010; Marques & Marandino, 2018; Sánchez et al., 2012; Silva & Pereira, 2019; Smith et al., 2021; Snellings et al., 2009).

Family support, access to resources, engagement in extracurricular reading activities, a stimulating social environment, well-prepared teachers, and robust school infrastructure play pivotal roles. These factors directly impact student learning outcomes (Babayigit et al., 2021; Gonçalves et al., 2013; Sampaio & Guimarães, 2007; Smith et al., 2021). Moreover, parental pressure for high-quality education and school administrations focused on market competition (Demo, 2007), further contribute to vocabulary acquisition. Vocabulary development is highly influenced by environmental factors and social interactions, shaping students’ linguistic competence (Gaskell & Ellis, 2009).

A study conducted in Brazil (Piccolo et al., 2016) analyzed the impact of Socioeconomic Status (SES) and parental education on the cognitive performance of children aged 6 to 12 in both public and private schools. The results revealed that SES and parental education, especially maternal education, had a significant influence on children’s performance in written and oral language. The acquisition of written language improves as the child grows and depends on interactions with the environment. Children from lower SES families face challenging environments with higher rates of crime and divorce. On the other hand, parents with higher educational levels tend to invest more in their children, providing access to books and educational resources. Early exposure to reading has a positive impact on language development, making children better prepared for school. Additionally, aspects of phonological awareness, such as the ability to recognize words, are also influenced by exposure to reading at home. Mothers with higher levels of education tend to read more to their children, positively affecting the development of written language. Therefore, socioeconomic and educational factors play a crucial role in the development of written and oral language in children.

The superior performance of private school students, when compared to public school students in the oral comprehension test, is in agreement with the low performance in reading comprehension tests. It is known that difficulties in reading comprehension may originate in oral language. It has a reciprocal relationship with the development of reading comprehension. The general ability to understand reading increases with reading experience and with spoken language, developing reciprocally with reading practice and experience (Cuetos, 2010; Morais, 2013; Perfetti et al., 2013; Sánchez et al., 2012; Smith et al., 2021).

Given these results, the answer to the initial question, “Will there be a difference in the evaluation of the reading processes between public and private elementary school students through tests of the Brazilian reading processes-PROLEC-SE-R?” is yes. Private school students have higher mean scores than public school students in word reading, showing that the use of orthography helps in reading processes. When the knowledge of the use of the word in text, extraction of meaning and its understanding was needed, the difficulty of access to the mental lexicon by the studied population became evident.

## Conclusion

Understanding the characteristics of schoolchildren who are no longer in the literacy cycle is extremely important. There exists an idealized image of what a proficient reader embodies, yet little emphasis is placed on evaluating the individual reading profiles of students within Brazilian pedagogical frameworks, particularly from Elementary School II onwards. Identifying where this process may encounter obstacles is paramount to fostering the implementation of specific strategies across educational levels, notably in primary and secondary education. Moreover, the PROLEC-SE-R tool not only delineates such reading profiles but also enables us to discern disparities in teaching and learning between public and private educational sectors during the literacy phase, which persist throughout the foundational stages of schooling.

## Abbreviations

CI: Confidence interval
CNPq: National council for scientific and technological development
CNS: National health council
G: Group
IFC: Informed consent form
INEP: Anísio Teixeira National Institute of Educacional Studies and Research
OECD: Organisation for Economic Co-Operation and Development
ENEM: National High School Examination
EJA: Youth and Adult Education
ANRESC: School Performance Evaluation
SES: Socioeconomic Status
p: *p* value
PISA: International Student Evaluation Program
PROLEC-SE-R: Reading Process Assessment tests
WR: Word Reading
PR: Pseudoword Reading
GSII: Grammatical Structures II
PM: Punctuation Marks
MRC: Mnemonic Reading Comprehension
PCR: Pure reading comprehension
LC: Listening Comprehension
SD: Standard Deviation
$$\overline{x}$$: (média)
